# Templating effect of clot structure can predict clot development and outcome in diluted blood: a comparison with thromboelastography

**DOI:** 10.1186/cc11036

**Published:** 2012-03-20

**Authors:** M Lawrence, J Kaczynski, S Stanford, R Morris, P Evans

**Affiliations:** 1 Swansea University, Swansea, UK; 2 ABMU LHB, Swansea, UK; 3 UWIC, Cardiff, UK

## Introduction

Treatment of major hemorrhage with colloids is known to have an effect on clot outcome. However, determining both the rate and extent of these changes is difficult. Development of a new biomarker has shown that it can detect structural development earlier and quantifies these changes to clot outcome accurately when compared to other methods. This study compares the fractal dimension, Df [1], found when the clot first forms to measures of mature clot firmness obtained from thromboelastography.

## Methods

Forty healthy blood samples were obtained; each sample was allocated a random dilution ratio (10%, 20%, 40%, 60%) and diluted with gelofusine. These were matched with 40 healthy samples that were undiluted. An oscillatory shear technique was applied to the blood using an AR-G2 measuring Df (clot structure). Additionally the clot development in terms of firmness was measured using a ROTEM analyser measuring at 5, 10, 15 minutes and its maximum (A5, A10, A15, MCF).

## Results

Df significantly decreases with increasing dilution. The decrease in structural complexity indicates that gelofusine even at 40% dilution is producing poor quality clots. See Table 1.

**Table 1:** Change in Df with dilution

Dilution %	Df
0	1.74 (0.05)
10	1.72 (0.04)
20	1.70 (0.06)
40^*^	1.63 (0.05)
60^*^	1.59 (0.06)

^*^Significant decrease from 0%.

## Conclusion

Df that is measured at the incipient clot is found much sooner than the mature clot parameters, between 5 and 30 minutes earlier. Df is significantly correlated (*P *< 0.05) with the mature clot parameters of clot firmness (A5, A10, A15 and MCF) and elasticity (G'_max_). This suggests that in dilution Df can determine the eventual clot outcome very early. Measurement of Df could guide fluid replacement and component therapy more accurately and earlier than conventional markers.
